# The influence of assisted reproductive technologies-related stressors and social support on perceived stress and depression

**DOI:** 10.1186/s12905-024-03262-1

**Published:** 2024-07-27

**Authors:** Aditi Gupta, Emily Lu, Zaneta Thayer

**Affiliations:** Department of Anthropology, Dartmouth College, Hinman Box 4036, Hanover, NH 03755 USA

**Keywords:** Infertility, Stress, Depression, IVF, ART

## Abstract

**Background:**

While assisted reproductive technologies (ART) have helped many people experiencing infertility become pregnant, the ART process can take a psychological toll. This study examined whether and how perceived stress- and depression-related symptoms vary among individuals at different stages of the infertility and ART process, and whether ART-specific stressors and emotional support are associated with mental health symptomatology.

**Methods:**

Data were collected using an online REDCap survey administered between July 2021 and March 2022. The survey was administered to 240 participants who had experienced infertility, including those who had not yet accessed ART, those undergoing ART but who were not yet pregnant, those currently pregnant through ART, and those who had given birth in the last year through ART. Each participant completed the Cohen Perceived Stress Scale (range 0–40) and the Edinburgh Depression Scale (range 0–30). Participants who had undergone ART were asked about their experience of ART-specific stressors and how helpful partner and provider support had been during the ART process. Survey data were analyzed using ANOVA and multivariate linear regressions.

**Results:**

88% of participants reported medium or high levels of perceived stress, and 43.8% of respondents showed probable indications of depression. Perceived stress and depression symptoms were significantly higher for individuals currently undergoing, but not yet pregnant from, ART treatments. These effect sizes were substantial; for example, depression scores in this group were five points higher than among currently pregnant individuals and nine points higher than among postpartum individuals. For the subset of participants who had used or were currently undergoing ART (*N* = 221), perceived social stigma and the physical and time demands of ART were significantly associated with higher stress and depression symptoms, while partner emotional support was associated with lower perceived stress.

**Conclusions:**

The ART process exacerbates perceived stress and depression symptoms among individuals experiencing infertility. Given the potential long-term impacts on both parent and child wellbeing, clinicians and policymaking groups, including the American Society for Reproductive Medicine (ASRM), should consider making access to mental health services a standard of care during infertility treatment.

**Supplementary Information:**

The online version contains supplementary material available at 10.1186/s12905-024-03262-1.

## Background

The number of assisted reproductive technologies (ART) cycles in the U.S. has more than doubled in the past decade [1]. In 2021 alone, 97,128 infants were born via ART, comprising more than 2% of all live births nationally [1]. While ART has been life-changing for many of the almost 10% of adult women and 9% of adult men who experience infertility [1, 2], aspects of the ART process are stressful and can negatively affect mental health.

Studies have shown that individuals who undergo ART procedures experience higher stress and pregnancy-specific anxiety than individuals who conceive spontaneously [3–5]. High levels of stress and anxiety among ART patients may reflect the trauma of treatment failure and the indefinite duration of infertility associated with undergoing ART [6]. Additional stressors include the financial burden of ART, uncertainty regarding pregnancy outcomes, and the physical pain stemming from ART treatments [7, 8]. Furthermore, a growing body of research aims to assess potential ways to mitigate the intensity of ART-related stressors. Specifically, studies have shown that counseling and partner support may reduce ART-related stress and depression [9–14].

Notably, singleton children conceived through ART are more likely to be born preterm and have lower birth weights [15]. These children are also more likely to experience adverse chronic health problems, such as cardiovascular disease, that may persist into adulthood [16, 17]. These health outcomes are commonly interpreted as the direct result of clinical ART procedures, such as the influence of media culture [18, 19] or the freeze-thaw embryo transfer process [20]. That said, a large body of research has demonstrated that maternal stress and anxiety during pregnancy are associated with similar patterns of birth outcomes in offspring, including lower birth weight [21, 22] and development of non-communicable diseases in adulthood [23]. Therefore, reducing ART-associated stressors is potentially important for improving parental mental health, optimizing birth outcomes, and improving longer-term offspring health.

This study aims to evaluate whether and how maternal stress- and depression-related symptoms vary across the infertility and ART process. We specifically predict that perceived stress and depression will be highest among individuals currently undergoing ART who have not yet become pregnant (Hypothesis 1). We then evaluate whether stressful aspects of infertility and ART that have been identified in previous literature – including the financial burden, psychological stressors (i.e., stigma, the uncertainty of outcome), and procedural logistics (i.e., time and physical demands) are associated with greater perceived stress and depression symptomatology (Hypothesis 2). Finally, we evaluate whether interpersonal support networks (i.e., partner and provider support) are associated with lower perceived stress and depression (Hypothesis 3).

## Methods

### Design

Data were collected from an online convenience sample using a REDCap survey between July 2021 and March 2022. Participants were eligible to participate if they were living in the United States and were currently experiencing infertility and considering ART, currently undergoing ART, currently pregnant through ART, or had given birth to a child via ART in the last year. We recruited survey participants through social media outreach to fertility clinics and infertility virtual support groups. A total of 359 participants completed the online survey. After accounting for missing variables, full data were available for *N* = 240 participants. This study received ethical approval from Dartmouth College (STUDY00032277), which relies on ethical principles outlined in the Belmont Report. Study participants provided informed consent through the survey.

Self-reported demographic information, including gender (female, male, gender non-conforming, non-binary/genderqueer, other: specified), age (years), race (White, Hispanic, Black, Asian, American Indian/Alaska Native, Pacific Islander, multiracial), household income (less than $10,000; $10,000 - $19,999; $20,000 - $34,999; $35,000 - $49,999; $50,000 - $74,999; $75,000 - $99,999; $100,000-$150,000; $150,000-250,000; $250,000+), and marital status (single, married/domestic partnership, divorced, widowed, separated) were collected.

### Dependent variables: mental health outcomes

#### Hypotheses 1–3: perceived stress and depression levels

Each survey participant completed the Cohen Perceived Stress Scale (PSS) [24] and the Edinburgh Depression Scale (EDS) [25]. Both the PSS and EDS have been widely used to measure perceived stress and depression, respectively, in perinatal populations and populations experiencing infertility [26–29].

The PSS is a 10-item survey asking participants about their emotions over the past month, with each question scored from 0 to 4. The developers state the PSS is an efficient and reliable indicator of stress and is highly correlated with life events, social anxiety, and psychosomatic symptomatology [24]. The maximum PSS score is 40; scores from 0 to 13 indicate low perceived stress, scores from 14 to 26 indicate medium perceived stress, and scores from 27 to 40 indicate high perceived stress.

The EDS is also a 10-item survey asking participants how they felt in the past 7 days, with each question scored from 0 to 3. The EDS is a highly sensitive and specific predictor of depression [25]. The maximum EDS score is 30 and scores over 13 are interpreted as indicating probable clinical depression [30].

#### Independent variables

##### Hypothesis 1: Infertility stage

Participants self-identified as being at one of four stages of the infertility process:

1. Individuals experiencing infertility and considering ART but who had not started treatment at the time of the survey.
2. Individuals currently undergoing ART but not pregnant at the time of the survey.
3. Individuals pregnant via ART at the time of completing the survey.
4. Individuals who had given birth to a child conceived using ART within the past year at the time of the survey.

##### Hypothesis 2: ART-related stressors

An original instrument was constructed based on previous qualitative studies to assess specific ART-related stressors in separate models. Participants were asked to indicate how stressful each of the following factors were while undergoing ART:

1. Their financial situation.
2. Uncertainty about ART success.
3. Shame about their infertility.
4. The physical demands of ART procedures (such as hormonal injections, egg retrieval, embryo transfer, etc.).
5. The time commitment for ART procedures (such as doctor appointments and travel).

Participants rated each potential stressor using a Likert scale from 1 (not stressful) to 5 (very stressful).

##### Hypothesis 3: Support systems

We asked participants how helpful different sources of support had been for their ART process using a Likert scale from 1 (not helpful) to 5 (very helpful). Sources of perceived support identified were “talking with partner” and “talking with your ART care provider.” These two variables were analyzed in separate models. Since the questions referred to ART-related support specifically, participants who were experiencing infertility but who had not started the ART process were not included in analyses related to social support. The sample size for these analyses was therefore *N* = 221.

### Statistical analysis

Survey data were downloaded from REDCap and analyzed in Stata version 17.0 and RStudio. Hypothesis 1 was evaluated by first conducting an ANOVA to test whether there were group differences in perceived stress and depression. If significant group differences were identified, multiple contrasts were explored using Tukey’s post-hoc tests. Study hypotheses 2 and 3 were evaluated using multivariate linear regressions. We analyzed separate models to determine whether sources of stress or support predicted perceived stress or depression, respectively. Regression analyses corrected for age, race, household income, marital status, and infertility stage. Alpha was set to 0.05 for all analyses.

## Results

### Summary statistics

We report descriptive statistics for the study sample in Table 1. The mean age of the respondents was 33.6 years (SD = 5.1 years). Almost half the participants (48.33%) indicated that they were undergoing but not currently pregnant via ART at the time of their response. All participants identified as female, and 92.9% of participants were married or in a domestic partnership. Sixteen participants (7.2%) were engaging with ART due to an interest in having a child with a same-sex partner. The majority (81.7%) of participants self-reported that they were White, and the median household income was between $100,000 and $150,000. The most reported out-of-pocket cost for ART was $30,000+ (22.2%).

**Table 1:** Summary of sample characteristics. Values represent N (%) for categorical variables and mean (SD) reported for continuous variables

		Total sample (*N* = 240)
Sample characteristics		
Age (years)		33.6 (5.1)
Self-identified race	White	196 (81.7%)
	Hispanic	9 (3.8%)
	Black	9 (3.8%)
	Asian	7 (2.9%)
	American Indian/Alaska Native	3 (1.2%)
	Multiracial	16 (6.7%)
Relationship status	Single	12 (5.4%)
	Married/domestic partnership	223 (92.9%)
	Divorced	2 (0.8%)
	Separated	3 (1.2%)
Household income	$10,000 - $19,999	2 (0.8%)
	$20,000 - $34,999	7 (2.9%)
	$35,000 - $49,999	17 (7.1%)
	$50,000 - $74,999	42 (17.5%)
	$75,000 - $99,999	51 (21.2%)
	$100,000-$150,000	66 (27.5%)
	$150,000-250,000	44 (18.3%)
	$250,000+	11 (4.6%)
Money spent on ART †	Less than $1,000	13 (5.9%)
	$1,000 - $2,999	12 (5.4%)
	$3,000 - $5,999	27 (12.2%)
	$6,000 - $9,999	31 (14.0%)
	$10,000 - $14,999	43 (19.5%)
	$15,000 - $19,999	15 (6.8%)
	$20,000-$30,000	31 (14.0%)
	$30,000 +	49 (22.2%)
Perceived stress score	0 (low) − 40 (high)	22.4 (7.2)
Depression score	0 (low) − 30 (high)	12.0 (5.6)

†*N* = 221

PSS and EDS scores indicated a high prevalence of stress and depression in the sample. 88% of participants reported medium or high levels of perceived stress (PSS score > = 14), while 43.8% of respondents showed probable indications of depression (EDS score > = 13).

#### Hypothesis 1: Perceived stress and depression symptoms are highest for individuals currently undergoing ART treatments

**Fig. 1:**
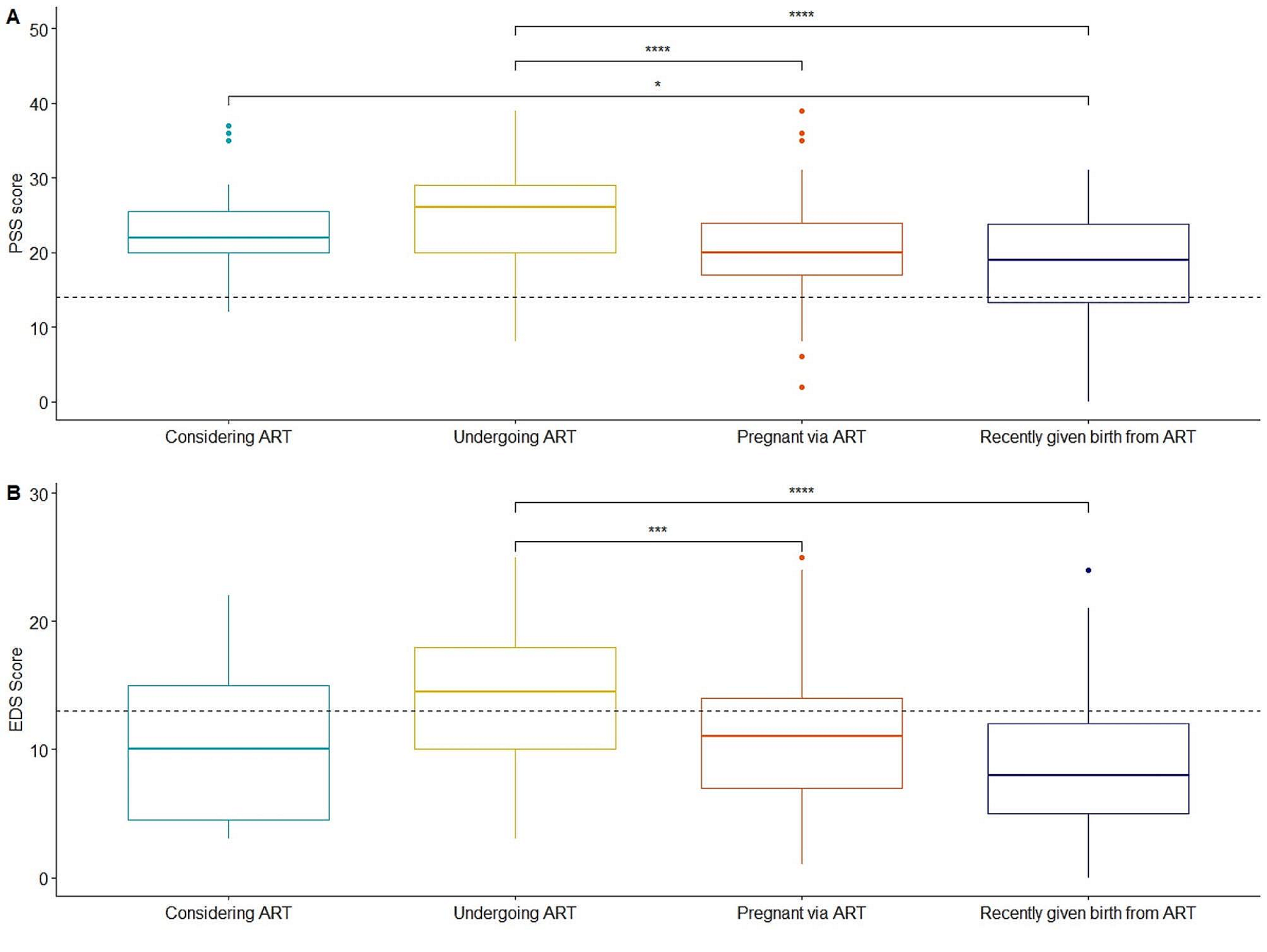
(**A**) Mean PSS scores for individuals significantly varied according to infertility stage. Associations significantly different in post-hoc tests are represented, with individuals currently undergoing ART having the highest mean PSS score. The line at the y-axis indicates the symptom threshold for moderate or high stress (PSS score > = 14). (**B**) Mean EDS scores for individuals significantly varied according to infertility stage. Associations significantly different in post-hoc tests are represented, with individuals currently undergoing ART having the highest mean EDS score. The line at the y-axis indicates the symptom threshold for probable depression (EDS score > = 13)

ANOVA tests demonstrated significant differences in perceived stress (F(3, 236) = [12.6], *p* < 0.001) and depression symptoms (F(3, 236) = [10.8], *p* < 0.0001) according to infertility stage (Fig. 1).

Consistent with predictions, post-hoc Tukey’s tests demonstrated that individuals currently undergoing ART had significantly higher perceived stress and depression scores than (1) individuals currently pregnant via ART (perceived stress: B = 4.46, *p* < 0.001; depression: B = 5.04, *p* = 0.004) and (2) individuals who had recently given birth via ART (perceived stress: B = 9.48, *p* < 0.001; depression: B = 7.20, *p* < 0.001). Individuals who had recently given birth from ART also had significantly lower perceived stress scores than those considering ART (B = 4.77, *p* = 0.046).

#### Hypothesis 2: ART-related stressors predict poorer mental health

##### Financial burden of ART

**Fig. 2:**
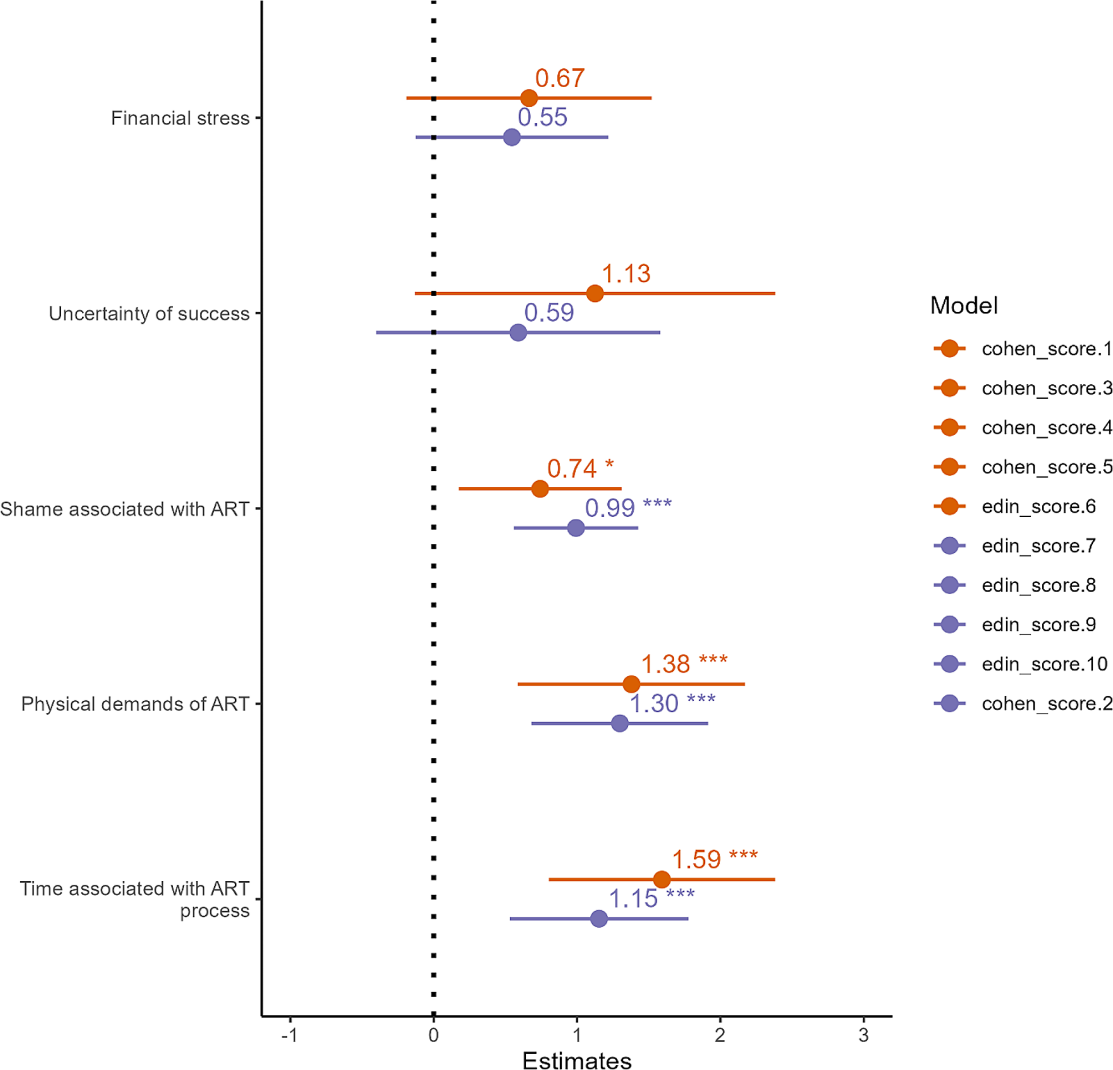
Effect size plot for ART-related stressors predicting perceived stress (orange) and depression (purple) scores in adjusted analyses. Infertility-related shame, the physical demands of ART, and the time associated with ART were all associated with significantly higher perceived stress and depression scores

The mean perceived financial stress, measured on a Likert Scale from 1 (not stressful) to 5 (very stressful), was 4.02 (SD = 1.08) (Suppl Table 1). While the associations were in the predicted direction, financial stress was not significantly associated with PSS (*p* = 0.125) or EDS (*p* = 0.11) (Fig. 2).

##### Psychological demands of ART

The mean reported stress associated with uncertainty about ART resulting in a pregnancy and/or live birth was 4.68 (SD = 0.72) (Suppl Table 1). Self-reported uncertainty about ART was not significantly associated with higher PSS (*p* = 0.08) or higher EDS scores (*p* = 0.23) (Fig. 2).

Perceived stress associated with shame about ART had a mean score of 3.09 (SD = 1.55) (Suppl Table 1). Shame-related stress was significantly associated with higher PSS (B = 0.74, *p* = 0.010, adj R^2^ = 0.20) and higher EDS scores (B = 0.99, *p* < 0.001, adj R^2^ = 0.24) (Fig. 2).

##### Procedural demands of ART

Greater reported physical demands of ART, such as the pain and/or invasiveness associated with hormonal injections, egg retrievals, and embryo transfers had a mean stress score of 3.91 (SD = 1.11) (Suppl Table 1). Higher stress associated with physical demands predicted significantly higher PSS (B = 1.38, *p* = 0.001, adj R^2^ = 0.22) and EDS scores (B = 1.30, *p* < 0.001, adj R^2^ = 0.23) (Fig. 2).

The time commitment associated with ART is a substantial burden associated with fertility treatment. The perceived stress of the amount of time spent on ART had a mean score of 3.90 (SD = 1.09) (Suppl Table 1). Greater reported stress associated with time demands of ART were significantly predictive of both higher PSS (B = 1.59, *p* < 0.001, adj R^2^ = 0.23) and EDS scores (B = 1.15, *p* < 0.001, adj R^2^ = 0.21) (Fig. 2).

**Fig. 3:**
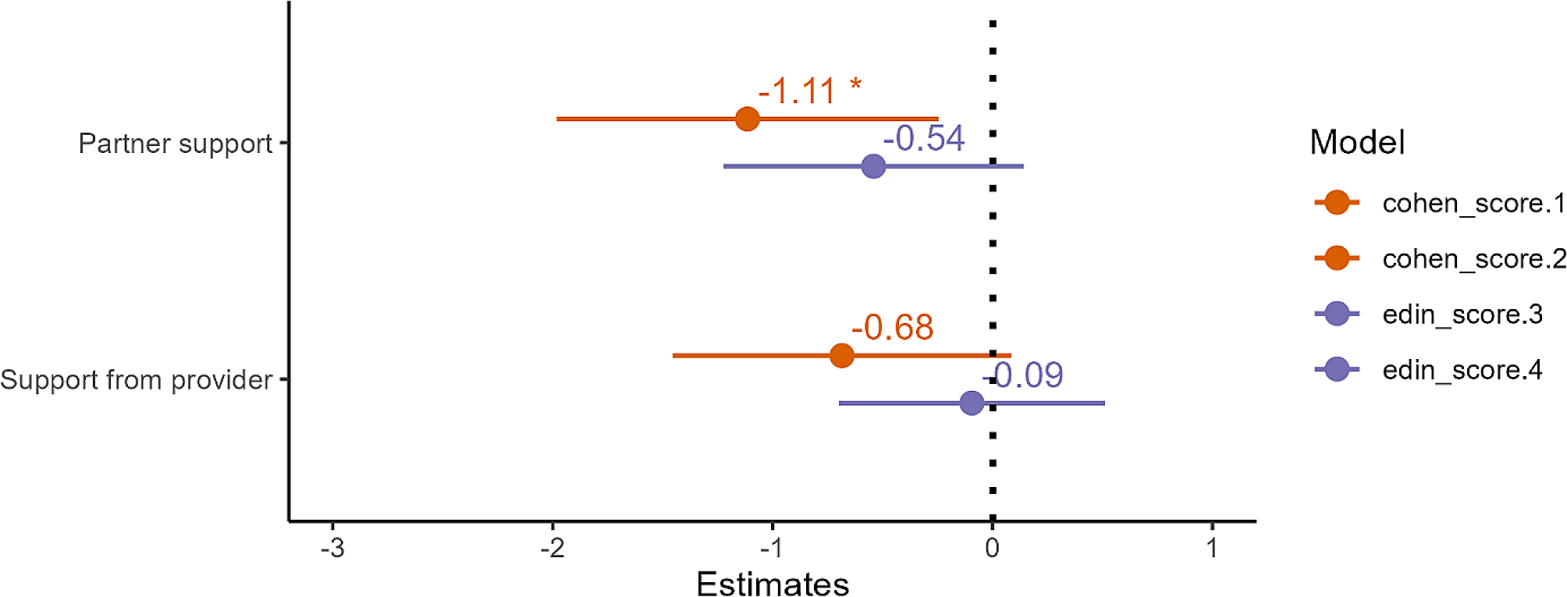
Effect size plot for social support predicting perceived stress (orange) and depression (purple) scores in adjusted analyses. Partner support, but not perceived support from one’s provider, was associated with significantly lower perceived stress

#### Hypothesis 3: Social support associated with less ART-related stress & depression

When rating the perceived benefit of talking with their partner about ART-related stress on a Likert score of 1 (not helpful) to 5 (very helpful), participants reported a mean score of 3.74 (SD = 1.17). Partner support was associated with significantly lower PSS scores (B = -1.11, *p* = 0.011, adj R^2^ = 0.20) (Fig. 3). The strength of the association with EDS was in the expected direction (B = -0.54) but did not reach statistical significance (*p* = 0.12) (Fig. 3).

Participants rated the mean perceived helpfulness of speaking with one’s provider about ART-related stress as 3.20 (SD = 1.22). Perceived support from one’s ART provider was not significantly associated with lower PSS (B = -0.68, *p* = 0.08) or lower EDS (B = -0.09, *p* = 0.76) (Fig. 3).

## Discussion

Our study demonstrates that psychological wellbeing varies across different stages of the infertility process and that ART-related stressors are associated with worse psychological wellbeing. While individuals across all stages of the infertility process reported moderate to high levels of perceived stress, individuals currently undergoing ART treatments reported the most perceived stress and depression symptoms. The mean EDS score for the group currently undergoing ART was above the clinical threshold, indicating probable depression. The psychological demands of ART, including social shame surrounding ART, were also predictive of adverse mental health outcomes. Finally, the physical pain and invasiveness of ART, and the time commitment to attend and travel to appointments, were also associated with higher perceived stress and depression symptoms. While we analyzed each stressor in individual models, in reality, ART-related stressors often co-occur (Suppl. Table 2) and can exacerbate one another.

### ART-related psychological stressors impact mental health

While infertility is inherently stressful, we found that individuals currently undergoing ART had the highest perceived stress and depression levels. The effect size was substantial; the EDS score, was, on average, a remarkable seven points higher for individuals currently undergoing ART compared to postpartum individuals. The perceived stress score was nine points different on average between the two groups. A post-hoc analysis demonstrated that the group currently undergoing ART also reported significantly more stress associated with the financial burden and shame from ART compared to the other groups (Suppl Table 1). These findings suggest that individuals undergoing ART are under immense psychological pressure, above and beyond that associated with infertility alone.

The stigma of infertility often leads to feelings of shame among individuals undergoing ART. Stress associated with shame was significantly associated with higher stress and depression scores in our analysis, with participants describing how infertility is often regarded as taboo and shunned by society. The time demands of undergoing ART, which were also associated with stress and depression, further compound the psychological burden of fertility treatment. Some patients must travel out of state or even abroad to seek cheaper fertility treatments, demonstrating higher financial and logistical burdens associated with ART procedures [31, 32]. Indeed, many individual financial factors can negatively affect one’s ART experience, including state residency and insurance conditions. The average cost of a single IVF cycle can range from $12,400 [33] to $25,000 [34], and the percentage of ART cycles that result in a live birth is only 37.2% [1].

The mean stress score associated with how ART affected participants financial situation was the second highest of all stressors assessed here. As of 2023, only 21 states mandate insurance to provide fertility coverage to qualifying residents, and only 15 of these laws cover IVF treatments [35]. Further, coverage varies by type of treatment service, and some states have monetary limits on covered costs. Mandated coverage is often limited to specific individuals; for example, some laws restrict eligibility to women of a certain age [36, 37]. Given the cost of ART, the low likelihood of a live birth per cycle, and varying insurance coverages, the financial burden of ART can be prohibitively expensive to pursue or continue.

Partner support was associated with significantly lower perceived stress in our analysis. The relationship between support from the ART provider and PSS was in the expected direction but did not reach statistical significance (*p* = 0.08); this could reflect a lack of a true association or limited statistical power.

#### Study recommendation: improving the standard of care for ART patients

Given the major societal, logistical, and financial stressors that ART poses, this study recommends increased insurance coverage of ART procedures and increased access to mental health services to mitigate ART-related challenges. Individualized and ART-specific counseling should be available for individuals and couples undergoing ART. Access to mental health services can mitigate some of the stressors identified by individuals in our study, such as feelings of anxiety and uncertainty during waiting periods.

Insurance companies would ideally cover fertility services equitably across the United States to reduce the stress faced by individuals undergoing ART. Universal insurance coverage mandates would require all group and individual health plans, public and private, to cover ART-related procedures and therefore decrease the financial stressors that exist at every stage of ART. More broadly, the logistic demands associated with ART, including the time to navigate insurance coverages, drive to clinics, and schedule repeated appointments, speak to a larger problem about the inaccessibility of healthcare for individuals across the United States [38, 39]. Improvements are needed in healthcare infrastructure and expanding healthcare access across spatial and socioeconomic lines.

Furthermore, previous research found that stress and financial costs were cited as the most common reasons for discontinuing IVF treatment [8]. Our study supports existing literature that ART-related stressors can adversely impact mental wellbeing; thus, it is not surprising that these experiences would lead to individuals stopping treatment. Through improved standards of care and access to mental health professionals, patients can better cope with the physical and psychological stressors during the ART process.

### Study limitations

This study consisted of a convenience sample of mostly well-educated, high-income individuals, which is commonly observed in online survey participation [40] and in populations seeking fertility treatment [41, 42]. Thus, this convenience sample only captures part of the spectrum of fertility experiences at different socioeconomic levels. As current estimates suggest less than a quarter of couples experiencing infertility can access the ART treatments they need, financial support could make treatment accessible to patients at all socioeconomic levels [43]. We also used original questions to assess Likert responses for both sources of stress and support to test Hypotheses 2 and 3. These stress and support factors were derived from prior qualitative research on individuals undergoing ART [9–14]. While we were able to compare the relationship between the financial stress variable and household income to assess construct validity (*r* = -0.23, *p* = 0.0005), we were unable to validate construct validity for the other measures. Additional psychometric evaluation is therefore needed to validate these results.

The sample varied in terms of reproductive stage, with both PSS and EDS used to assess stress and depression, respectively, in all groups. This was done so that individuals differing in reproductive status could be assessed using the same measures. However, it’s worth noting that the clinical cut-point for depression, though not used analytically in this study, may vary across reproductive stages [30]. In addition, the moderately small sample sizes (*N* < 100) in the groups considering ART, currently pregnant via ART, and recently given birth to a child conceived through ART reduced the statistical power of the regressions and ANOVA tests. Furthermore, regressions that analyzed social support exclude responses from those who had not yet started the ART process. Additional studies are therefore needed in larger samples to replicate these findings.

## Conclusion

The roller coaster of unsuccessful treatments really effected [sic] my mental health…And, while I am thrilled to finally be pregnant, I know I will carry the trauma of what it took to get me here for the rest of my life. (Study participant who was pregnant via ART at the time of survey)

While ART has provided undeniable progress for individuals experiencing infertility, there are aspects of the ART experience that are extremely stressful and sometimes overlooked by care providers primarily concerned with achieving a viable pregnancy and birth. This study explored variation in perceived stress and depression symptoms across different stages of the infertility process, and the impact of specific ART-related stressors on perceived stress and depression. Our findings show that individuals experiencing infertility and undergoing ART procedures reported high levels of stress and depression. Individuals currently undergoing ART treatment had the highest stress and depression symptoms. Perceived social stigma and the physical and time demands of ART procedures were associated with higher stress and depression symptoms, while partner support was associated with lower perceived stress among these individuals.

Additional research is needed to understand whether ART-related stress is associated with negative physical health outcomes in mothers and their offspring. For example, the Developmental Origins of Health and Disease (DOHaD) hypothesis would suggest that ART-associated psychosocial stress experienced during pregnancy can lead to an increased risk of poor offspring health across their life course [44–46]. As an important next step, future research should evaluate how mental stress during ART affects longer term infant health. Additionally, our study corrected for social factors (specifically age, race, household income, and marital status) to better evaluate how ART-related stressors impact patient stress and depression independent of these factors. That said, future research should critically explore how these and other social determinants of health—including gender and religious beliefs—shape how patients experience infertility-related stress and stigma. By identifying how social factors shape ART-related stress, providers can better support individual patients undergoing fertility care. Finally, the American Society for Reproductive Medicine (ASRM) regards a fertility/mental health counselor as an auxiliary, but recommended, component in ART clinics [43]. While not explored specifically in this analysis, counseling with mental health professionals (MHPs) at ART facilities may alleviate psychosocial stressors associated with treatment. In addition, MHPs working at the facility will have increased knowledge of the specific treatments offered, promoting a greater understanding of a patients’ emotional needs. Making MHPs mandatory could provide a beneficial source of support for individuals undergoing ART and should be explored in future research.

### Electronic supplementary material

Below is the link to the electronic supplementary material.

Supplementary Material 1

## Data Availability

The datasets generated and/or analyzed during the current study are not publicly available due to privacy concerns associated with the sensitive nature of the research. De-identified data are available from the corresponding author with appropriate ethical review approval.
