# Supplementary material for: The influence of assisted reproductive technologies-related stressors and social support on perceived stress and depression
Source: BMC Womens Health. 2024 Jul 27;24:431. doi: 10.1186/s12905-024-03262-1 (PMC11282751; doi:10.1186/s12905-024-03262-1)
Supplement: Supplementary file 1 — Supplementary Material 1 [file 12905_2024_3262_MOESM1_ESM.docx]

Supplementary Table 1. Summary scores reported degree of stress associated with ART procedures and helpfulness of speaking about ART-related stress with others according to infertility / ART stage. Scores for stress variables are on a scale from 1 (not stressful) to 5 (very stressful), while the social support variables are on a scale from 1 (not helpful) to 5 (very helpful). P-value represents result of ANOVA test.

|  | **Considering ART** | **Pregnant via ART** | **Recently Given Birth from ART** | **Undergoing ART But Not Pregnant** | **Across All Groups** | **p** |
| --- | --- | --- | --- | --- | --- | --- |
|  |  |  |  |  |  |  |
| **Stress variables** |  |  |  |  |  |  |
| Financial stress | 4.5 (0.7) | 3.8 (1.1) | 3.7 (1.3) | 4.1 (1.0) | 4.0 (1.1) | 0.002 |
| Uncertainty of success | 4.6 (0.7) | 4.5 (1.0) | 4.7 (0.6) | 4.7 (0.7) | 4.7 (0.7) | 0.199 |
| Shame associated with ART | 3.3 (1.7) | 2.8 (1.4) | 2.7 (1.5) | 3.3 (1.5) | 3.1 (1.6) | 0.03 |
| Physical demands of  ART | 3.7 (1.6) | 4.0 (1.0) | 4.1 (1.0) | 3.8 (1.1) | 3.9 (1.1) | 0.361 |
| Time associated with  ART process | 3.6 (1.4) | 3.9 (0.9) | 3.8 (1.1) | 4.0 (1.1) | 3.9 (1.1) | 0.599 |
| **Support with ART process variables** |  |  |  |  |  |  |
| Partner support | - | 3.7 (1.2) | 4.0 (1.2) | 3.6 (1.2) | 3.7 (1.2) | 0.248 |
| Support from provider | - | 3.4 (1.2) | 3.5 (1.3) | 3.0 (1.2) | 3.2 (1.2) | 0.01 |

Supplementary Table 2. Correlation matrix representing correlation among individual ART-related stress variables.

           Uncertainty  Shame  Physical  Time  Financial

Uncertainty       1.00   0.13      0.16  0.24       0.07

Shame             0.13   1.00      0.20  0.25       0.11

Physical          0.16   0.20      1.00  0.42       0.02

Time              0.24   0.25      0.42  1.00       0.18

Financial         0.07   0.11      0.02  0.18       1.00
